# Spotlight on early-career researchers: Interview with Gerrit Maus

**DOI:** 10.1038/s42003-018-0130-7

**Published:** 2018-09-03

## Abstract

Gerrit Maus began his independent career at Nanyang Technological University in October 2015. In this short interview, part of our series highlighting early-career researchers (https://www.nature.com/articles/s42003-018-0061-3), he tells us about his experience as an early-career researcher, the advice he would give to his younger self, and the amazing superpowers of our eyes.

**Figure:**
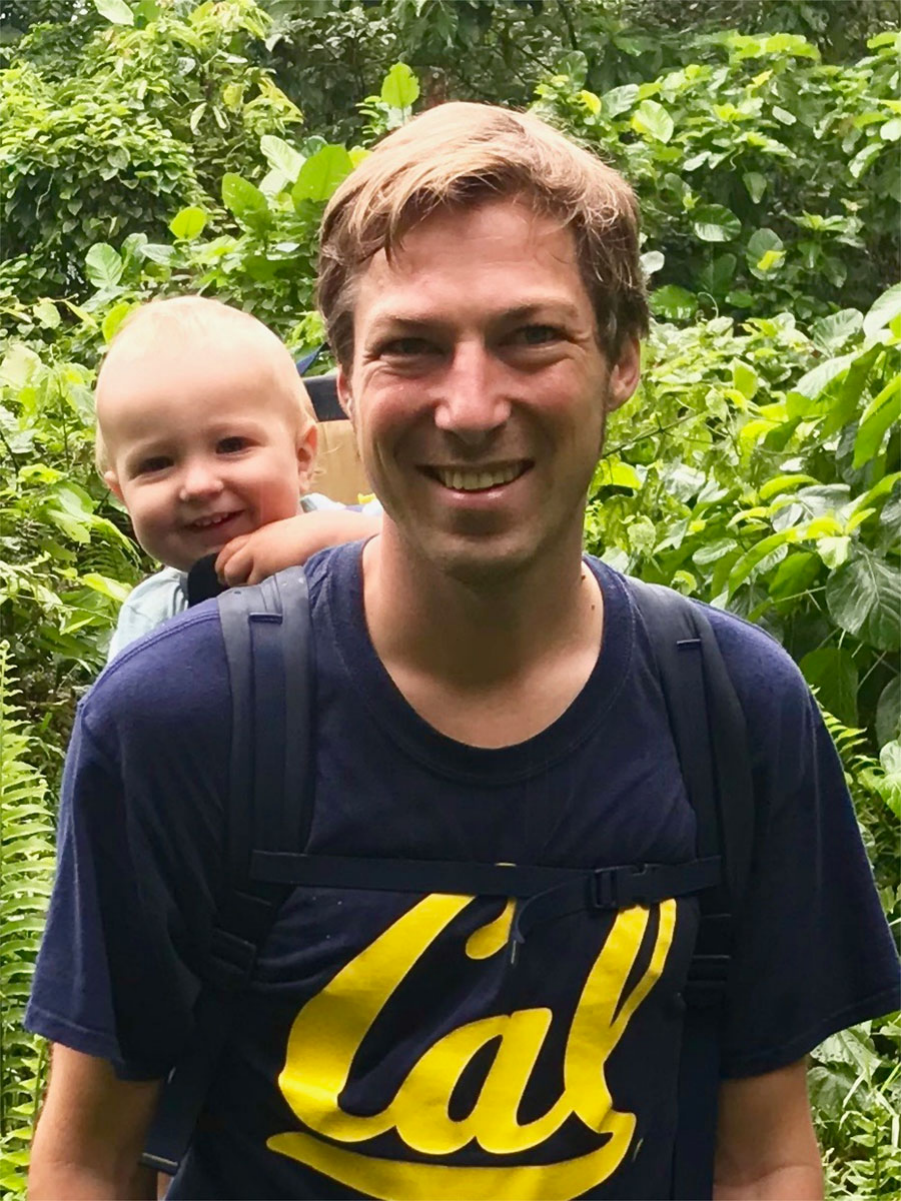
Gerrit Maus

**Please tell us about your research interests**

The input coming in from the eyes does not contain complete information about our environment, yet we usually have the impression that we perceive a complete “picture” of our surroundings. For example, we are not perturbed by the lack of resolution or colour in the periphery, the disruption of input during eye blinks, the lack of signals from the retinal blind spots, or the inevitable delays of all bottom-up input before signals arrive in cortical areas. Our brain seems to happily “make up” data to compensate for what’s missing or delayed. I am interested in understanding how visual perception deals with missing or delayed information, and how extrapolation and interpolation mechanisms in the human brain work to fill in these gaps in space and time.

**What has your journey been to this point?**

I chose a rather broad and interdisciplinary academic education which, in retrospect, benefitted me a lot. As an undergraduate, I studied Cognitive Science at the University of Osnabrück in Germany. The programme combined diverse topics in Computer Science and Artificial Intelligence, Cognitive Psychology and Neuroscience with some emphasis in Linguistics, Philosophy and Mathematics. Thus, from the start I never experienced strict boundaries between disciplines. In addition, the programme had a large proportion of students and lecturers from overseas and most classes were taught in English, which certainly made the transition to living and working in other countries easier.

I decided to pursue a Ph.D. in Cognitive Psychology at the University of Sussex in England after a spending a semester abroad in that same institution. Once I graduated, I moved to California to work with David Whitney, first at University of California Davis and then at Berkeley. Although this was originally planned to be a 2-year or 3-year postdoc, it quickly turned into 7 years and funding was scraped together from various sources… sometimes contracts only lasted 3 months!

The job market in our field is insanely competitive and I applied for positions all over the world for several years before accepting the offer for my current position at Nanyang Technological University in Singapore. Coming to Singapore has been a great choice because the scientific environment is impressive, there are a lot of resources, relatively easy ways to secure funding and productive labs doing world-class science in many fields. While the bureaucracy can feel stifling at times, there is a can-do spirit in Asia that may be declining in other parts of the world.

**What are your predictions for your field in the near future?**

Vision science as a field is quickly becoming more technical, more data-heavy and more “neuro”. Many studies combine data from multiple imaging modalities and computational modelling. All of these developments hold great promise to make real steps forward in understanding how vision works at a mechanistic level in the human brain. I also think, however, that it is important not to forget the phenomenological side of things. What do we actually see, what does it feel like to perceive something? After all, we are (or at least I am) interested in explaining human experience, and without studying the perceptual content with carefully chosen stimuli and tasks, much of the neuroscience data may be meaningless to answer these questions. The best research will be that which successfully combines behavioural, neuroimaging and modelling approaches.

**Can you speak of any challenges that you have overcome?**

First, I would like to state that as a white middle-class male I am sure my challenges are minimal compared to what others from different backgrounds face.

An ongoing personal challenge for me is how to be a good scientist, husband and father—all at the same time. The expectation of early-career researchers is still too often that they sacrifice everything for their career, to get that next paper published, to get that grant or that job offer. However, events in your life surely influence how much you are willing to invest in that career. For example, the birth of our son last year has made me feel conflicted about how to spend my time. Whenever I was at work, I felt the strong urge to be by my newborn’s side; and when I stayed home, I panicked feeling I was not doing enough for my career. The irony here is that the academic career path, unlike other career paths, in theory allows for a healthy and flexible management of time spent working. However, career and peer pressure, whether real or perceived, may take that advantage away, often with disastrous consequences for people’s happiness or their willingness to pursue science as a profession. I believe we hold the power to change our culture from within by leading by example—which is what I am trying to do now.

A related challenge has been how to balance mine and my partner’s career. We are committed to an equal partnership, sharing equally not only our household and childcare duties, but also the choice of our next geographical location. I have been fortunate that my partner’s career is less geographically constrained than mine—but still, finding a location where we can both have fulfilling careers has not been easy. Luckily, Singapore right now is that location for us.

**What advice would you give to your younger self?**

Don’t be afraid to surround yourself with people that are smarter than you! It’s daunting, and you feel stupid most of the time, but it’s the only way to grow smarter yourself.

Teaching is fun, but only if you take it seriously. If you don’t, you and your students will suffer.

Don’t fret if Plan A doesn’t work! Be flexible, move on to Plan B (and C, and D, and …)!

**What eye-related superpower would be the most useful to have?**

I think the powers that our eyes and brains equip us with are already pretty amazing; like being able to perceive the colour or surface properties of objects in a large range of illumination conditions, or being able to hit a fast moving tennis ball, although it is moving at a speed that makes the information arriving in our brain lag behind by metres, or even being able to tell hundreds of individuals apart and read their emotions by just looking at their face which—let’s face it—all look pretty similar. But, if I had to choose an additional superpower I would probably go for being able to sense a wider range of the electromagnetic spectrum, like Geordi La Forge’s “VISOR” from Star Trek. Seeing additional wavelengths would open up some pretty cool new experiences; seeing infrared, for example, would allow you to spot camouflaged animals and improve our detection of human emotions, or you might be able to see new “alien colours”.

*This interview was conducted by Associate Editor Yomayra F. Guzmán*

